# Real‐time fetal brain tracking for functional fetal MRI

**DOI:** 10.1002/mrm.29803

**Published:** 2023-07-19

**Authors:** Sara Neves Silva, Jordina Aviles Verdera, Raphael Tomi‐Tricot, Radhouene Neji, Alena Uus, Irina Grigorescu, Thomas Wilkinson, Valery Ozenne, Alexander Lewin, Lisa Story, Enrico De Vita, Mary Rutherford, Kuberan Pushparajah, Jo Hajnal, Jana Hutter

**Affiliations:** ^1^ Centre for the Developing Brain, School of Biomedical Engineering & Imaging Sciences King's College London London UK; ^2^ Biomedical Engineering Department, School of Biomedical Engineering & Imaging Sciences King's College London London UK; ^3^ MR Research Collaborations Siemens Healthcare Limited Camberley UK; ^4^ CNRS, CRMSB, UMR 5536, IHU Liryc Université de Bordeaux Bordeaux France; ^5^ Institute of Neuroscience and Medicine 11, INM‐11 Forschungszentrum Jülich Jülich Germany; ^6^ RWTH Aachen University Aachen Germany; ^7^ Department of Women & Children's Health King's College London London UK; ^8^ MRI Physics Group Great Ormond Street Hospital London UK

**Keywords:** BOLD, diffusion MRI, fetal brain development, fetal MRI, motion correction, motion detection, T2* relaxometry, tracking

## Abstract

**Purpose:**

To improve motion robustness of functional fetal MRI scans by developing an intrinsic real‐time motion correction method. MRI provides an ideal tool to characterize fetal brain development and growth. It is, however, a relatively slow imaging technique and therefore extremely susceptible to subject motion, particularly in functional MRI experiments acquiring multiple Echo‐Planar‐Imaging‐based repetitions, for example, diffusion MRI or blood‐oxygen‐level‐dependency MRI.

**Methods:**

A 3D UNet was trained on 125 fetal datasets to track the fetal brain position in each repetition of the scan in real time. This tracking, inserted into a Gadgetron pipeline on a clinical scanner, allows updating the position of the field of view in a modified echo‐planar imaging sequence. The method was evaluated in real‐time in controlled‐motion phantom experiments and ten fetal MR studies (17 + 4‐34 + 3 gestational weeks) at 3T. The localization network was additionally tested retrospectively on 29 low‐field (0.55T) datasets.

**Results:**

Our method achieved real‐time fetal head tracking and prospective correction of the acquisition geometry. Localization performance achieved Dice scores of 84.4% and 82.3%, respectively for both the unseen 1.5T/3T and 0.55T fetal data, with values higher for cephalic fetuses and increasing with gestational age.

**Conclusions:**

Our technique was able to follow the fetal brain even for fetuses under 18 weeks GA in real‐time at 3T and was successfully applied “offline” to new cohorts on 0.55T. Next, it will be deployed to other modalities such as fetal diffusion MRI and to cohorts of pregnant participants diagnosed with pregnancy complications, for example, pre‐eclampsia and congenital heart disease.

## 1 INTRODUCTION

### 1.1 Motivation

The fetal period is characterized by a range of rapid and intricate developmental processes. Deviations in this orchestrated cascade of events are linked to pathology and pregnancy complications such as congenital heart disease,^1^ pre‐eclampsia,^2^ and fetal growth restriction^3^ among others. While ultrasound imaging remains the first‐line screening modality, fetal MRI plays an increasing role in both research and clinical settings. In addition to the high spatial resolution provided, the soft tissue contrast, the reduced operator dependence, the ability to image until late gestation, and the availability of a range of functional contrasts are significant benefits. Particularly important among these functional techniques is T2* relaxometry, which relies on the Blood‐Oxygen‐Level‐Dependency (BOLD) effect to provide a proxy measure of oxygenation in the fetal brain^4^ and placenta.^5^ T2* has been shown to be decreased in the placenta of pre‐eclamptic pregnancies,^2^ pregnancies affected by congenital heart disease^6^ and those with fetal growth restriction^7^ among others. Furthermore, diffusion MRI (dMRI), can sensitise the imaging to tissue microstructure.^8^

Fetal motion is uncontrollable, often large, and unpredictable and poses one of the main challenges for these techniques, especially in early and mid‐gestation when fetuses have enough space for significant motion, and it affects all parts of the imaging and analysis process. With more precise and sophisticated functional techniques available to reveal ever finer details, precise correction of motion artifacts is growing in importance. Examples of this are BOLD imaging, T2* relaxometry and diffusion MRI, with increasing resolution, higher b‐values, and dynamic information. Such techniques rely on the acquisition of the same slice stack location multiple times to then be combined for quantification and are particularly susceptible to motion which hampers robust analysis. Additionally, signal saturation (T1 recovery) issues cannot be corrected with postprocessing techniques.

Prospective acquisition correction techniques already exist for the enhancement of BOLD imaging with real‐time automatic head motion correction during the acquisition. Fetal motion detection and correction are, however, significantly more challenging than ex‐utero as a result of (1) larger displacements and rotations due to the inability to restrain and reduce motion, (2) the unpredictability of the motion, and (3) the surrounding uterine environment and maternal tissue. Typically, rapid single‐shot acquisitions are chosen to freeze the motion during the acquisition of each slice. The inter‐volume motion is then often corrected with postprocessing solutions such as registration‐based^9^ or signal‐model approaches.^10^ However, prospective techniques allow reacting in real‐time and thus enhance data quality immediately. The reduction or elimination of changes of pose in the imaging coordinates, applied during the scan, would lead to higher quality data—and hence a better starting point for post‐processing. Additionally, it could allow for the diffusion gradients to be adapted in real‐time and constitute a crucial step to address signal variation induced by motion and improve spatiotemporal analysis.

### 1.2 Related work

Fetal brain segmentation is the fundamental initial step in fetal motion correction. It is particularly challenging and crucial due to (1) the arbitrary position of the fetus within the uterus and (2) fetal motion. Machine‐learning‐based techniques have been successfully applied in various postprocessing settings to identify and segment the fetal brain. However, the vast majority are applied to high‐resolution anatomical scans based on fast spin‐echo and not echo‐planar imaging (EPI)‐based sequences, which typically display reduced resolution, quality (e.g., B0 inhomogeneities, geometrical distortions such as “ghosting”) and altered intensities/contrast.

Keraudren used bundled scale‐invariant feature transform features to automatically extract a bounding box around the fetal brain^11^ and segmented the brain using an approach based on random forests and conditional random field.^12^ Both methods were applied to two‐dimensional (2D) slices of high‐resolution acquisitions, with an average computation time for brain localization of <1 min and an additional 2 min for brain extraction in,^12^ thus not suitable for real‐time applications. Taimouri^13^ proposed an atlas‐matching technique aligning the brain rigidly. Tourbier^14^ presented an atlas‐based approach to extract the brain using a predefined bounding box. This method uses deformable registration to multiple atlases and is therefore also computationally very expensive. Fast fully automated segmentation of the intracranial volume was achieved in Reference 15, with the proposal of a voxelwise CNN adopted from Reference 16. Dice metrics slightly above 90% were reported for all the above‐mentioned techniques. Real‐time fetal brain extraction was first achieved^17^ with the use of a 2D UNet and a voxelwise fully convolutional network for segmenting the fetal brain in each 2D fetal MRI slice in about 1 second. Although this fetal brain extraction technique is significantly faster than the methods above, it cannot be applied to the task in this study as it relies on brain localization in each individual 2D slice. Instead of the entire three‐dimensional (3D) slice stack. Uus proposed an automatic simultaneous localization of both fetal brain and trunk^18^ to be applied to quality assessment of the images in real‐time by combining a 3D UNet and a discriminator module.^19^

The technique by McDaniels,^20^ using frame‐to‐frame registration in volumetric navigators, was extended by Gagoski by integrating EPI‐based volumetric navigators in the HASTE acquisition scheme.^21^ Gholipour proposed symmetric diffeomorphic deformable registration for the construction of a four‐dimensional atlas of the developing fetal brain,^22^ however, this method is computationally very expensive for a real‐time application.

There is, indeed, a gap between retrospective implementations and clinical applications. These implementations are often unsuitable for deployment in clinical/research clinical environments. The development of the above‐mentioned fetal brain motion tracking algorithms is typically done for "offline" applications, thus integration of such algorithms in MRI scanners to be applied "online" is challenging. To improve the capture range of subject‐to‐atlas registration, Salehi proposed the use of deep regression CNNs trained to estimate the 3D pose of the fetal brain based on image slices and volumes with the potential for real‐time applications.^23^ In Reference 24, a fetal pose estimation method was presented using deep learning algorithms to detect key fetal landmarks, achieving an average error of 4.47 mm and 96.4% accuracy. A state‐of‐the‐art novel deep predictive motion tracking framework was proposed in^25^ to address dynamic, real‐time 3D fetal brain motion using estimation and prediction of the motion trajectory based on MRI slice time series. Gagoski^21^ developed a state‐of‐the‐art acquisition/reconstruction pipeline that prospectively detects poor‐quality fetal brain HASTE images using a semi‐supervised image quality assessment CNN and automatically reacquires the stacks. Although the aim of both presented methods is aligned with the aim of our work, instead of high‐resolution HASTE images we apply our method to intrinsically lower‐resolution EPI images that will simultaneously be used for estimating head motion and analysing the brain in BOLD MRI and dMRI.

Benner^26^ proposed a motion correction and re‐acquisition method for adult brain diffusion‐weighted imaging, involving (1) calculation of rigid transformation with real‐time to adjust the next acquisition and (2) immediate re‐acquisition of the images with the highest real‐time calculated artefact level score. A correction strategy applied to diffusion‐weighted images by Dubois in Reference 27 relies on (1) automated detection and 2D resampling of the outlier individual slices and (2) 3D registration and realignment of misregistered volumes. To improve renal BOLD imaging, Morrell^28^ proposed continuous respiratory navigation and real‐time feedback for renal free‐breathing BOLD MRI.

### 1.3 Contributions

This study presents an intrinsically motion‐robust deep‐learning‐based fetal MRI method to achieve real‐time (intra‐scan) fetal head position and motion estimation and update the acquisition geometry prospectively to address the challenge of fetal brain motion in functional fetal MRI. Our method uses Gadgetron^29^ for real‐time reconstruction of the scans and a deep learning network adopted from^18^ for extremely fast fetal head localization. Intrinsic navigator images (EPI) are acquired—the target scan is simultaneously used for detecting the changes in head position and monitoring the fetal brain in BOLD MRI. Unlike the state‐of‐the‐art work presented in Reference 25, with real‐time estimation of the fetal head motion parameters, our method not only extracts the translational displacements as it also corrects them in real‐time in the following repetitions of the scan. Furthermore, contrary to the method presented in Reference 21, the field of view (FOV) adjustments that correct for the fetal brain motion are applied prospectively while the sequence runs, thus it does not require re‐acquisition and avoids extending the duration of the scan. The results presented are on single‐echo gradient‐echo EPI acquisitions, the foundation for most functional techniques used in fetal imaging. Thus, this work paves the way for future developments in diffusion and functional MRI.

## 2 METHODS

BOLD images are acquired and used as navigators, allowing for both spatiotemporal analysis of the BOLD signal and extracting the fetal brain motion parameters. Once acquired, the data is exported during the scan to Gadgetron, reconstructed, and brain localization is performed. Motion is estimated and sent from the Gadgetron server to the scanner, with the reconstructed slice stack, to adapt the FOV parameters of the following acquisition.

### 2.1 Feedback setup

**FIGURE 1:**
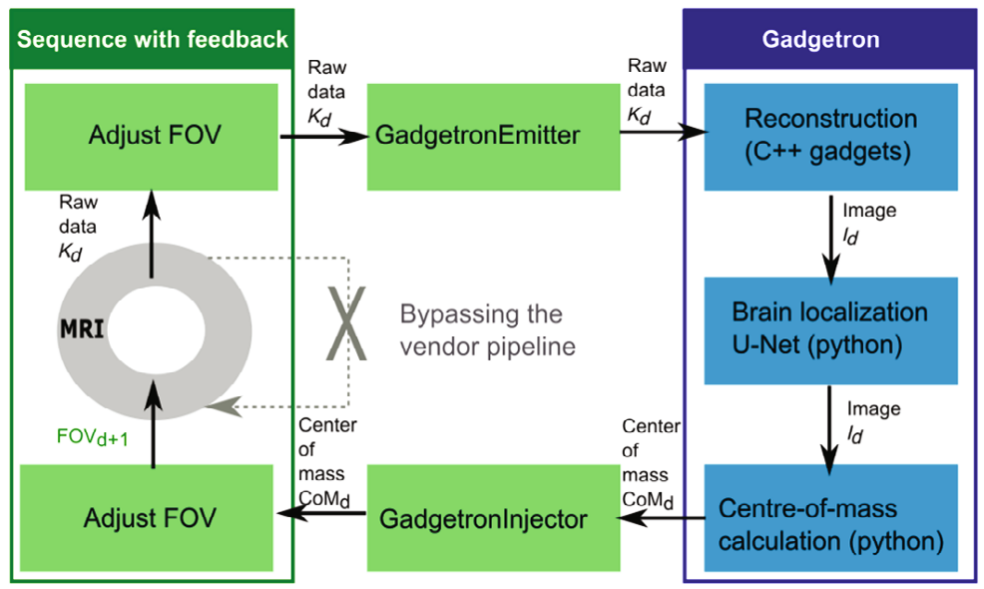
Schematic overview of the complete pipeline illustrating data acquisition, image reconstruction, fetal brain localization, and the real‐time change in the field of view (FOV).

The motion tracking and correction process was implemented on a clinical 3T scanner (MAGNETOM Vida, Siemens Healthcare). An intrinsic navigator approach was chosen where the target EPI scans are simultaneously used to detect the changes in head position and to obtain the functional contrast of interest. The complete setup is schematically illustrated in Figure 1, highlighting the changes performed both on the acquisition and reconstruction side. A gradient‐echo single‐shot multi‐dynamic EPI sequence was modified to receive updates for the FOV and to apply these translational changes in phase‐ and frequency‐encoding directions and slice selection to subsequent repetitions. On the reconstruction side, a Gadgetron pipeline^29^ was deployed on an external GPU‐equipped (NVIDIA GEFORCE RTX 2080 Ti, NVIDIA Corporate, Santa Clara, CA) computer which was connected to the internal network of the MRI scanner. This involves conversion of the raw data to ISMRMRD format immediately during the scan and reconstruction with off‐the‐shelf Gadgets that provide generic building blocks for configuring the streaming reconstruction in the Gadgetron. Then, a Python Gadget was written to estimate the position of the target object in the image. Two options were implemented: an intensity‐based segmentation method for phantom experiments as well as a pretrained 3D UNet^18^ to extract the fetal brain location and the center‐of‐mass (CoM) at each time point. There is currently some latency in the method that causes the FOV adjustment to be applied two repetitions after motion occurs, therefore the translational displacement of the CoM between time points *n* and n−2 was stored in the dynamic image header, sent back to the scanner with the respective image *n*, and accessed by dedicated feedback functors that send the motion parameters to the sequence to adapt the centre of FOV in the next repetition.

### 2.2 Localization network

#### 2.2.1 Network architecture

**FIGURE 2:**
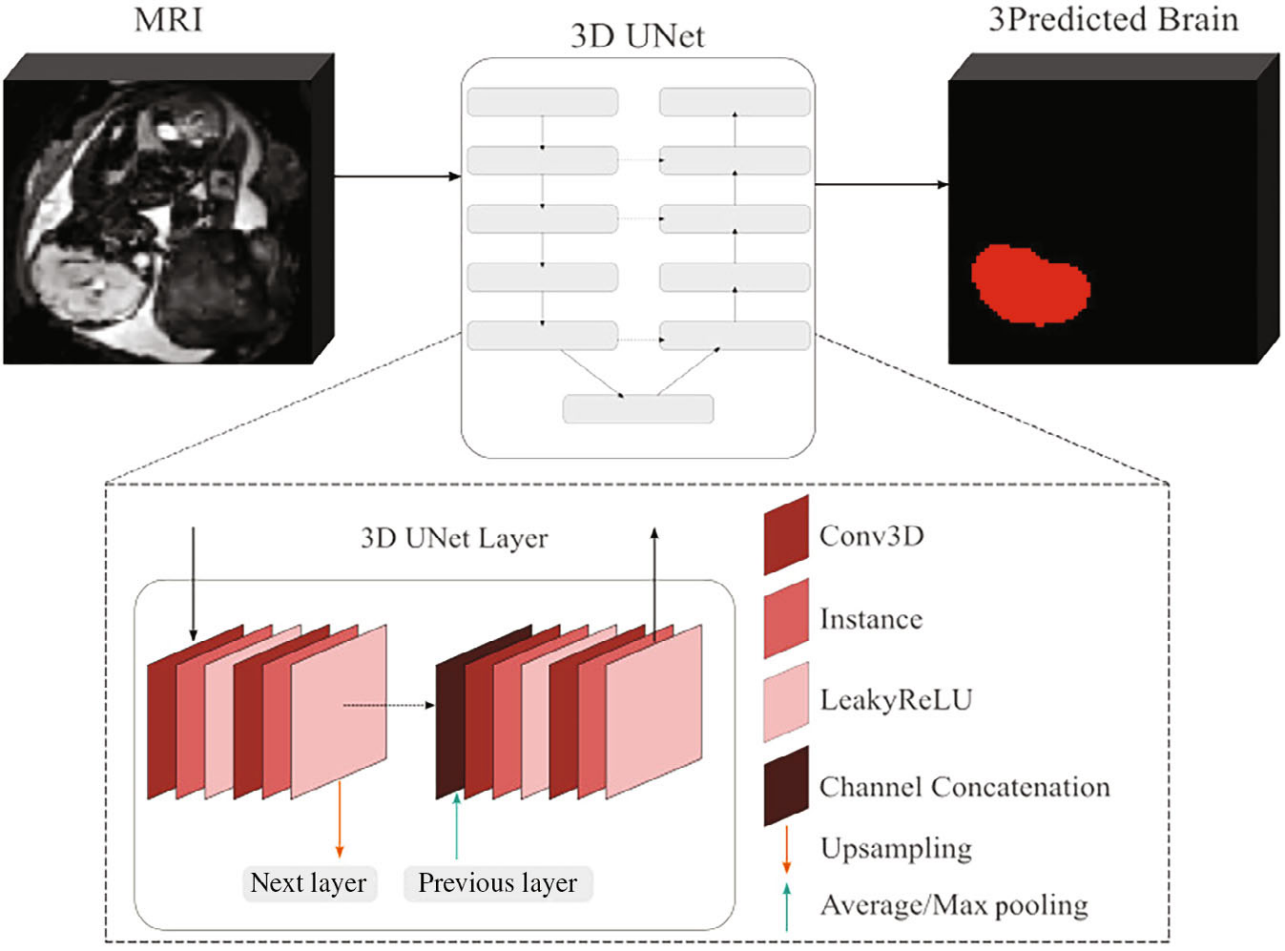
Pipeline for localization of fetal brain in MRI images.

The baseline architecture of the above‐mentioned network is a 3D UNet as in Reference 18 (Figure 2). It comprises five encoding‐decoding branches with 32, 64, 128, 256, and 512 channels, respectively. Each encoder block consists of 2 repeated blocks of 3×3×3 convolutions (with a stride of 1), instance normalization,^30^ and LeakyReLU activations. The first two down‐sampling blocks contain 2×2×2 average pooling layers, while the others use 2×2×2 max pooling layers. The decoder blocks have a similar architecture as the encoder blocks, followed by upsampling layers. The model outputs an *N*‐channel 3D image, corresponding to our *N* = 2 classes: background and fetal brain.

The network is trained by minimising the generalized Dice loss (GDL)^31^ (Equation 1) using the Adam optimizer with the default parameters ($\beta_{1}$ = 0. 9 and $\beta_{2}$ = 0.999).

(1) $$L_{GDL}=1-2\frac{\sum_{k=1}^{M}w_{k}\sum_{n}p_{kn}t_{kn}}{\sum_{k=1}^{M}w_{k}\sum_{n}p_{kn}+t_{kn}},$$

where $w_{k}=1/{\left(\sum_{n}t_{nk}\right)}^{2}$ is the weight of the *k*th tissue type, *p*_kn_ is the predicted probabilistic map of the *k*th tissue type at voxel *n*, *t*_kn_ is the target label map of the *k*th tissue type at voxel *n*, and *M* is the number of tissue classes. The network was implemented in PyTorch and the TorchIO^32^ library was used for data augmentation.

#### 2.2.2 Datasets, acquisition, and preprocessing

The dataset for training, validation, and initial testing consists of 157 scans acquired at 1.5 and 3 T, and it was split into 125 (training), 16 (validation), and 16 (test) scans. The trained model was, in addition, applied to two different datasets: (1) 29 subjects scanned at 0.55T to retrospectively evaluate the generalization ability of the model; (2) 10 fetal subjects scanned at 3T where the model was applied to the images prospectively and brain masks were generated upon each repetition of the scan.

The data used for the training and network performance evaluation includes gradient‐echo multi‐echo single‐shot EPI scans of 157 fetal subjects deliberately varying in field strength (1.5T/3T), echo time (TE; 7.9–240.2 ms), resolution (2/2.5/3 mm^3^ isotropic), acceleration factor (none, 2, 3), gestational age (15–40 weeks), fetal health (control cases, fetal growth restriction, prolonged preterm rupture of the membranes, etc.), and fetal position (cephalic, breech, transverse) to increase the robustness of the network. An additional test set including scans of 29 fetal subjects (17.6–36.3 gestational weeks) acquired at 0.55T was used to assess the performance of the trained model in low‐field MR images. The following protocols were used: 1) 3T Philips Achieva, 32‐channel cardiac coil and 16‐channel spine coil, matrix size =144×144−192×192, resolution =2×2×2/3×3×3 mm^3^, TEs =3.8/70.4/127/183.6 ms /10.1/54.3/98.4/142.5/186.8 ms, slices = 45–75; 2) 1.5T Philips Achieva, 28‐channel torso coil, matrix size = 144×144−288×288, resolution = 1×1×1/2.5×2.5×2.5 mm^3^, TEs = 14.6/77.4/140.1/202.8/265.5 ms, slices = 30–96; (3) 0.55T Siemens MAGNETOM Free.Max, blanket‐like BioMatrix Contour‐L 6‐element coil and fixed 9‐element spine coil, matrix size = 100×100−128×128, resolution = 3.1^3^–4.0^3^, TEs = 46/120/194/268/342 ms, slices = 50–59. Gold standard 3D brain masks were manually drawn for each of these datasets. Slice stacks and corresponding brain masks were resampled using padding and cropping to fit a 128×128×128 voxel grid matrix.

The prospective work here presented includes gradient‐echo single‐echo single‐shot EPI scans of 10 fetal subjects acquired on a clinical 3T Siemens MAGNETOM Vida scanner using an 18‐channel UltraFlex Large body coil and a 32‐channel spine coil. The imaging parameters applied were TR = 11 500 ms, matrix size = 148×148, resolution = 3.0 mm^3^, 15–34 repetitions, slices = 56–68, TE = 90 ms. Gestational age ranged from 17.2 to 34.7 weeks (mean 28.05 ± 5.86 weeks).

These scans were acquired as part of several ethically approved studies (16/LO/1573, 07/H0707/105, 17/LO/0282, 08/LO/1958, and REC19/LO/0852). The demographics and various acquisition parameters used are given in Figure S1.

#### 2.2.3 Training and network performance evaluation

The model was trained on images from 125 fetal subjects, validated on 16, and performance was evaluated on a test set of 16 subjects. This distribution approximates a ratio of 80%:10%:10% and ensures the range of acquisition settings, gestational ages and fetal positions present during training and testing are similar for an unbiased performance evaluation. Out of the 157 datasets, 45 were added to increase the representation of the two under‐represented classes, A) breech position and B) fetuses of gestational age <23 weeks. Class A) adds up to 37% of all fetal subjects and B) to 22%, and the representation of these two classes in training/validation/testing datasets was kept at similar percentages.

The localization performance was analyzed with the Dice similarity coefficient (DSC) and Intersection‐over‐Union (IoU) evaluation metric calculated between the gold standard manual segmentations performed by fetal MRI experts (LS, JH, SNS, and JAV with respectively 12, 9, 2, and 1 year[s] of experience) and the segmentations obtained from the network.

To assess the effect of the computed DSC on the estimation of fetal head motion, images from one fetal subject of the test set (3 TEs, 30 repetitions) were used. Manual segmentations were drawn and the localization network was used to generate predicted masks across 3 TEs for the first 10 repetitions of the scan. Mean squared errors (MSE) were computed between the CoM coordinates of the ground‐truth and predicted brain masks.

### 2.3 Experiments

#### 2.3.1 Phantom experiments

The method was evaluated first in real‐time on a 3T Siemens MAGNETOM Vida scanner using a spherical glass phantom filled with manganese‐chloride‐doped agarose to achieve relaxation properties similar to the fetal brain. The imaging parameters applied were TR = 6740 ms, voxel size = 3.0 mm^3^, 50 repetitions, 40 slices, TE = 90 ms. Motion was mimicked with controlled rectangular translational displacements throughout the acquisition in x, y, and z directions (from right to left, bottom to top, and front to back of the inside of the bore). The accuracy of the FOV changes was evaluated by comparing the CoM coordinates at the beginning of the scan (first repetition) and the CoM in repetitions where motion correction was applied.

#### 2.3.2 Fetal experiments

Fetal data were acquired from 10 pregnant volunteers in St Thomas' Hospital recruited between October 2022 and February 2023 after informed consent was obtained as part of an ethically approved study (MEERKAT, REC19/LO/0852, Dulwich Ethics Committee, December 8, 2021). Women were scanned on the above‐described clinical 3T Siemens Vida scanner in supine position. Fetal head motion was measured and corrected prospectively with real‐time adjustments of the acquisition geometry.

## 3 RESULTS

### 3.1 Localization task

**FIGURE 3:**
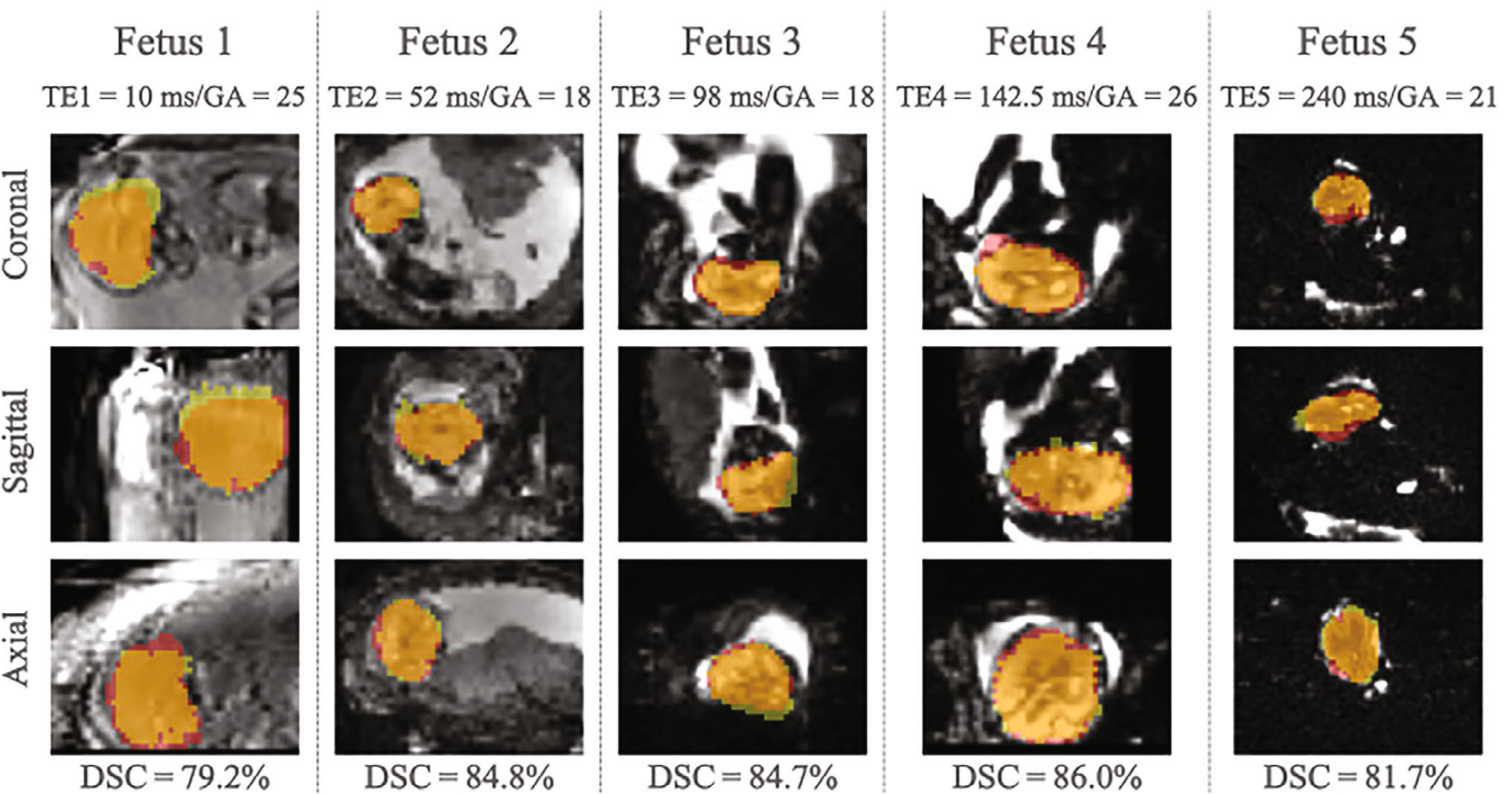
Images of five fetal subjects (25/18.6/15.7/26/21 gestational weeks) obtained at five echo‐times (10/52/98/142.5/240 ms) with brain masks predicted by the network (red) and corresponding manually drawn masks (yellow).

The process of brain localization took between 11.43–20.85 ms per volume in testing mode “offline.” Figure 3 shows examples of predicted brain masks generated by the 3D‐UNet (red), the corresponding ground‐truth segmentations (yellow), and the overlap between the two (orange).

**FIGURE 4:**
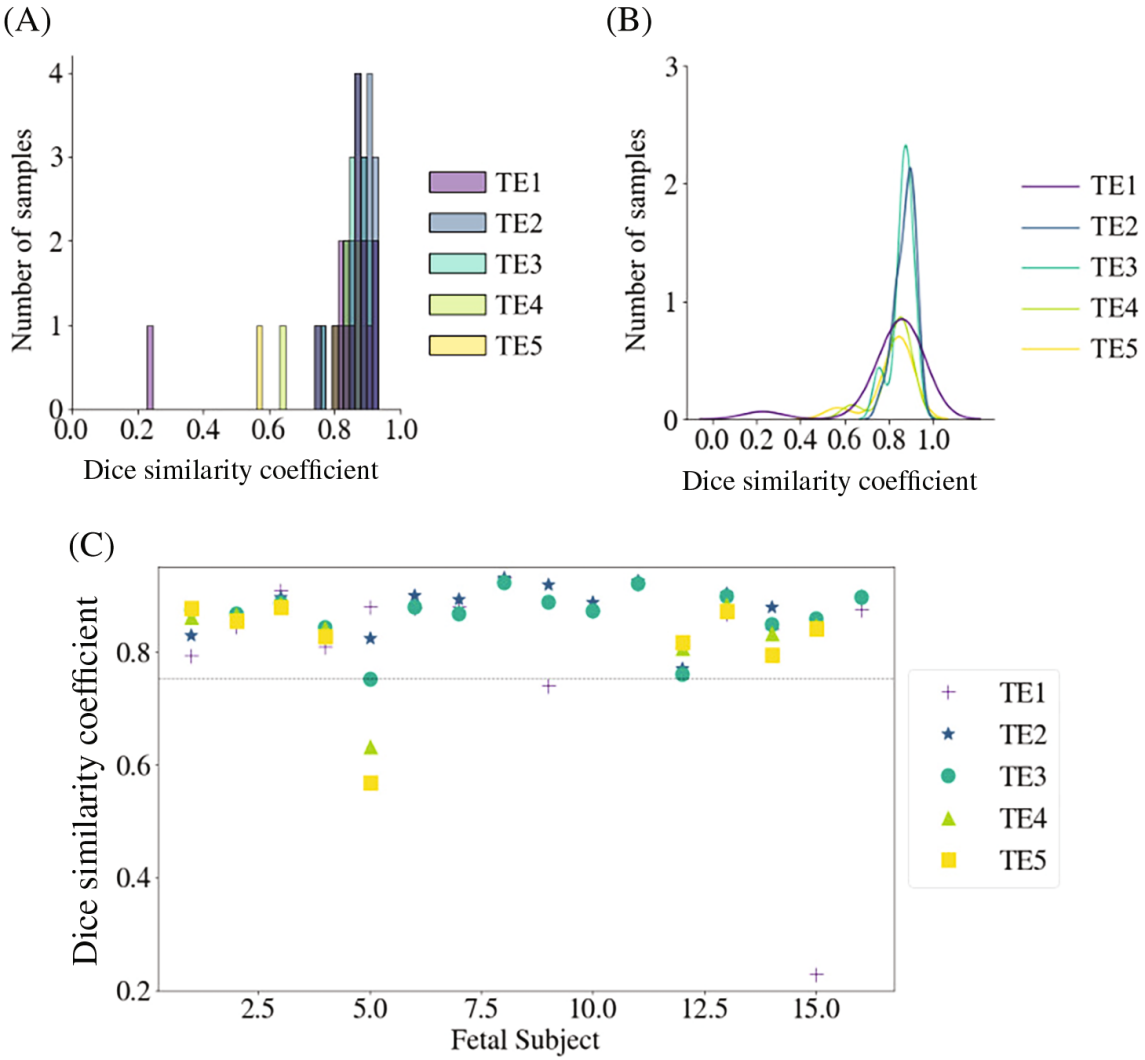
(A) Histogram of Dice similarity coefficient results obtained from brain masks automatically extracted from images of 16 fetal subjects at five echo times. (B) Density plot representing the distribution of Dice coefficient scores across five echo times. (C) Dice coefficient values were calculated and plotted individually for each fetus at each echo time.

A histogram (Figure 4A) and a density plot (Figure 4B) show the distribution of the DSC results computed for the 16 fetal subjects comprised in the test set for each TE. In the first TE, DSC results ranged between 22.8% and 93.1%, with values above 80% for 14 subjects. DSC values increased in the second and third TEs, with results ranging between 77.0%–93.0% and 75.2%–92.2%, respectively. DSCs exceeded 80% in 15/14 fetal subjects in TE2/TE3, respectively. Of the 16 fetuses, nine fetuses were scanned using five TEs, in which DSC results decreased to 63.3%–88.5% in TE4 and 56.9%–87.9% in TE5. Overall, the best performance was observed in TE2 and the lowest in TE5.

**FIGURE 5:**
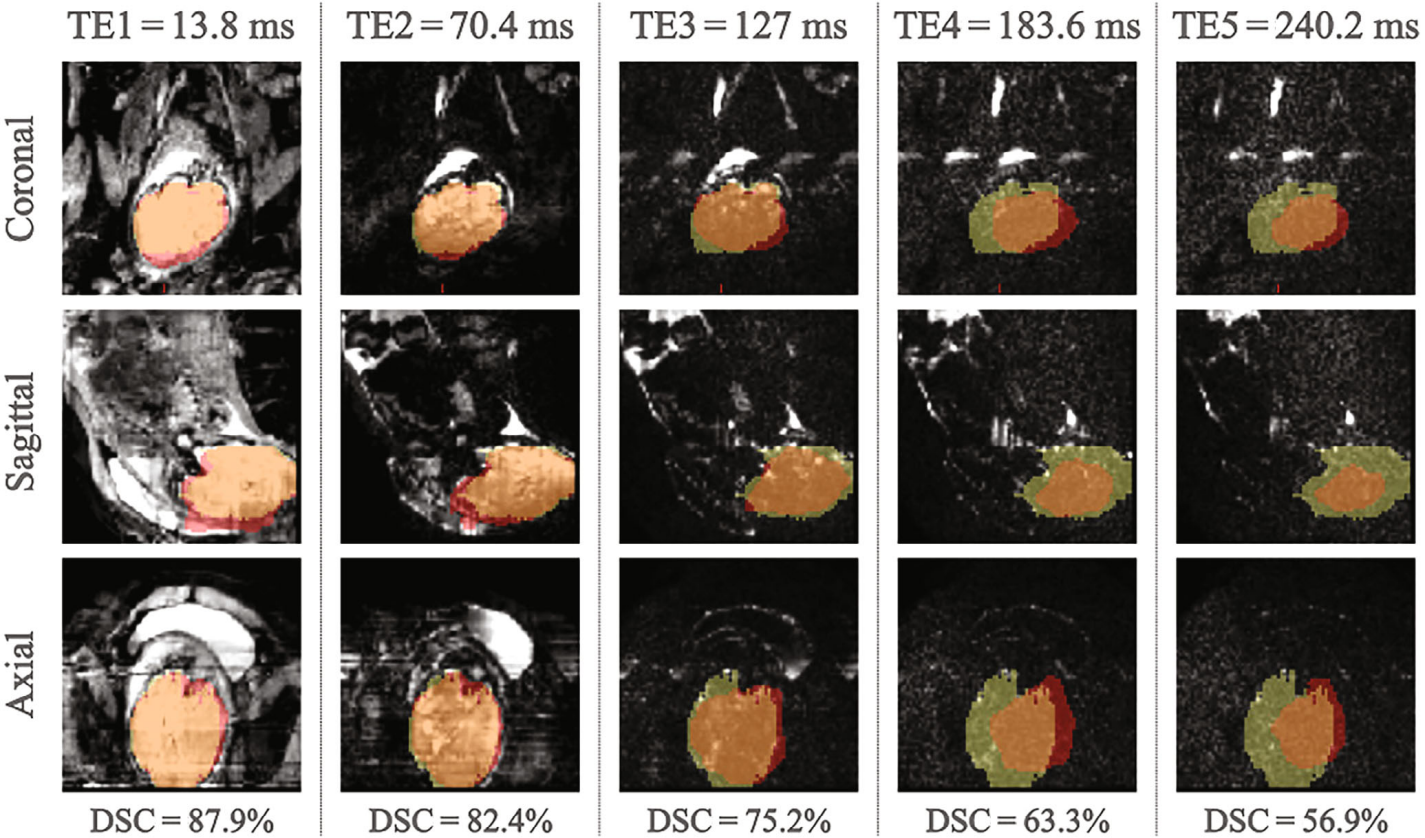
Images of Fetus 5 at TE1/TE2/TE3/TE4/TE5 = 13.8/70.4/127/183.6/240.2 ms with respective ground‐truth (yellow) and predicted (red) brain masks.

The DSC values individually calculated for each TE image of each subject are plotted in Figure 4C. Fetal subject 5 showed the highest variance in DSC across the five TEs, with values of 75.2% and above in TE1/TE2/TE3, followed by a significant drop to 63.3% and 56.9% in TE4 and TE5, respectively. High variance was also observed in subject 9, with a significant increase from TE1 (74.0%) to TE2 (92.0%) and TE3 (88.9%). Fetus 15 initially scored a DSC of 22.8% in TE1 and increased significantly to above 84.1%. DSC values for all other fetal subjects and all TEs remained above the 75% threshold (see Figure 4C). Figure 5 demonstrates the signal loss and image artefacts associated with the decreasing performance of the model with an increase in TE on the ground‐truth (yellow) and predicted (red) brain masks.

All results are summarised in Table 1. The trained model achieved overall DSC and IoU performances of 84.4 ± 9.8% and 73.5 ± 10.3%, respectively, across all TEs, fetal positions, and gestational ages. Regarding TE, the model performance initially increased with an increase in TE, associated with the improved contrast provided by such images. Comparable performance was achieved in TE2 and TE3. DSC and IoU decreased in TE4, with TE5 showing the lowest performance. To assess the model performance on different fetal positions, the test set was divided into two classes‐ breech (seven subjects) and cephalic (nine subjects). Slightly higher performance was observed in the cephalic position (higher by DSC = 3.3%, IoU = 4.1%). Lastly, the test set was divided into two classes according to gestational age—below and above 23 gestational weeks with 5 and 11 subjects, respectively. Slightly lower performance was observed in the <23 GA class (lower by DSC = 3.8%, IoU = 4.4%).

**TABLE 1:** Mean Dice coefficient values calculated between ground‐truth and network‐predicted fetal brain masks for two test sets, one consisting of (A) 16 fetal subjects scanned at 1.5/3T between 15 and 40 gestational weeks, and (B) 29 fetal subjects scanned at 0.55T between 17.6 and 38.1 weeks gestational age, evaluated for echo‐time (TE), fetal position, and gestational age.

Echo‐time					
	TE1	TE2	TE3	TE4	TE5
1.5/3T					
DSC (%)	81.8 ± 15.9	87.5 ± 4.3	86.4 ± 4.6	82.8 ± 7.3	81.5 ± 9.1
IoU (%)	71.6 ± 17.2	78.0 ± 6.5	76.4 ± 6.9	71.3 ± 9.5	69.6 ± 11.3
0.55T					
DSC (%)	83.1 ± 19.0	84.8 ± 16.7	83.2 ± 16.0	79.1 ± 17.2	79.8 ± 18.0
IoU (%)	74.2 ± 19.2	76.2 ± 17.7	73.6 ± 17.1	68.1 ± 19.0	69.5 ± 20.8

Fetal position		
	Breech	Cephalic
1.5T/3T		
DSC (%)	82.5 ± 12.3	85.8 ± 7.0
IoU (%)	71.6 ± 13.2	75.7 ± 9.4
0.55T		
DSC (%)	83.3 ± 9.3	82.0 ± 19.0
IoU (%)	72.3 ± 11.5	72.7 ± 20.2

Gestational age		
	≤23	>23
1.5/3T		
DSC (%)	82.0 ± 13.2	85.6 ± 7.1
IoU (%)	71.1 ± 13.7	75.4 ± 9.6
0.55T		
DSC (%)	71.5 ± 22.2	84.0 ± 16.0
IoU (%)	59.7 ± 23.3	74.7 ± 17.1

Abbreviations: DSC, dice similarity coefficient; IoU, intersection‐over‐union.

The CoM calculated from ground‐truth and predicted brain masks from TE1/TE2/TE3 images were plotted for one fetal subject for the first 10 repetitions of the multidynamic scan, as illustrated in Figure S3. The average DSC of the predicted brain masks for TE1 was 74.1%, 90.1% for TE2, and 86.7% for TE3, across 10 repetitions. MSE were computed between the CoM coordinates of the ground‐truth and predicted masks. In TE1, MSE was [5.64, 545.0, 0.13] ± [9.22, 458.29, 0.097] mm in x, y, z, respectively. For TE2, MSE was [0.85, 0.72, 0.47] ± [0.76, 1.24, 0.46] mm. MSE in TE3 was [3.2, 61.14, 0.69] ± [8.08, 182.29, 0.42] mm. Motion estimation thus failed in TE1 due to segmentations in the stomach region in addition to the brain for all repetitions except 5 and 7. Similarly, the stomach was also segmented in repetition 6 for TE3. Lower DSC in TE1 did not affect the motion estimation in x and z significantly, however, motion estimation in y failed. Although the overall DSC in TE3 was lower than in TE2 by 3.4%, the motion was successfully estimated for all repetitions but 6. As expected, TE2 provided the lowest mean MSE and the fetal head displacement estimation was therefore the closest to the ground‐truth motion detection.

#### 3.1.1 Fetal brain localization in low‐field MRI scans

**FIGURE 6:**
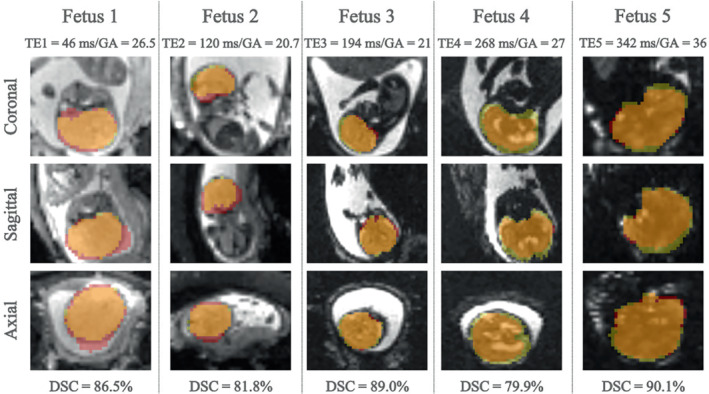
Images of five fetal subjects (26.5/20.7/21/27/36 gestational weeks) obtained at 0.55T and at 5 echo‐times (46/120/194/268/342 ms) with brain masks predicted by the network (red) and corresponding manually drawn masks (yellow).

When tested on low‐field (0.55T) MR images, the model trained on 1.5T/3T datasets achieved an overall DSC of 82.3 ± 17.5% and IoU of 72.6 ± 18.8% across all TEs, fetal positions, and gestational ages. Figure 6 shows examples of predicted brain masks generated by the 3D‐UNet (red) and the corresponding ground‐truth segmentations (yellow).

Table 1 summarizes all results. Regarding TE, the best performance was achieved in TE2, followed by TE1 and TE3 where comparable performance was observed. DSC and IoU values decreased in TE4. TE5 presented the lowest score according to the DSC, whereas TE4 showed the lowest performance using the IoU evaluation metric. The model performance was also assessed according to fetal position‐ breech (six subjects) and cephalic (23 subjects), and gestational age‐ ≤23 gestational weeks (four subjects) and above 23 weeks (25 subjects). Comparable performance was observed for the two fetal positions, with the cephalic position outperforming breech (DSC = 1.5%, IoU = 0.5%). The class of fetal subjects with GA >23 weeks outperformed the class GA ≤23 weeks (DSC = 12.5%, IoU = 15.0%).

#### 3.1.2 Real‐time fetal brain localization

**FIGURE 7:**
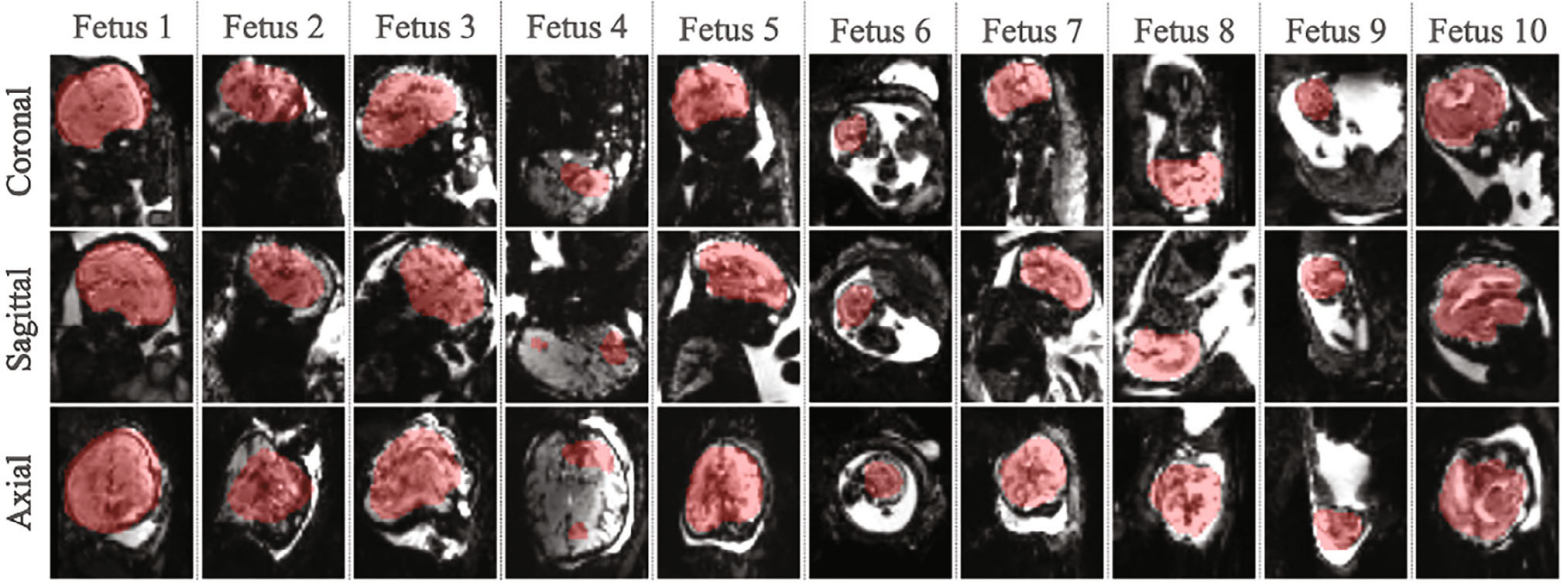
Coronal, sagittal and axial views with regard to the brain from all ten prospectively acquired fetal datasets illustrating the automatically achieved segmentation in red.

Figure 7 illustrates the achieved prospective localization in real‐time for all 10 fetuses. A volume approximate to the fetal brain volume was extracted for all but one fetal subject (Fetus 4).

### 3.2 Real‐time fetal brain tracking

#### 3.2.1 Phantom experiments

**FIGURE 8:**
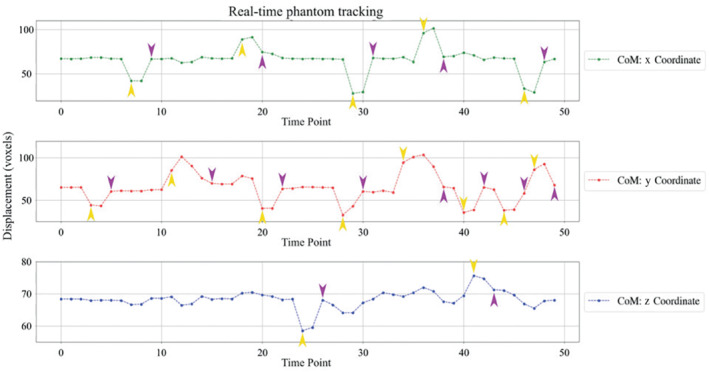
The calculated center‐of‐mass coordinates in all three axes are depicted for the phantom experiment. Yellow arrows indicate the detected motion, and purple arrows the corresponding resulting automatic Field‐of‐View correction as applied two repetitions after the movement.

**FIGURE 9:**
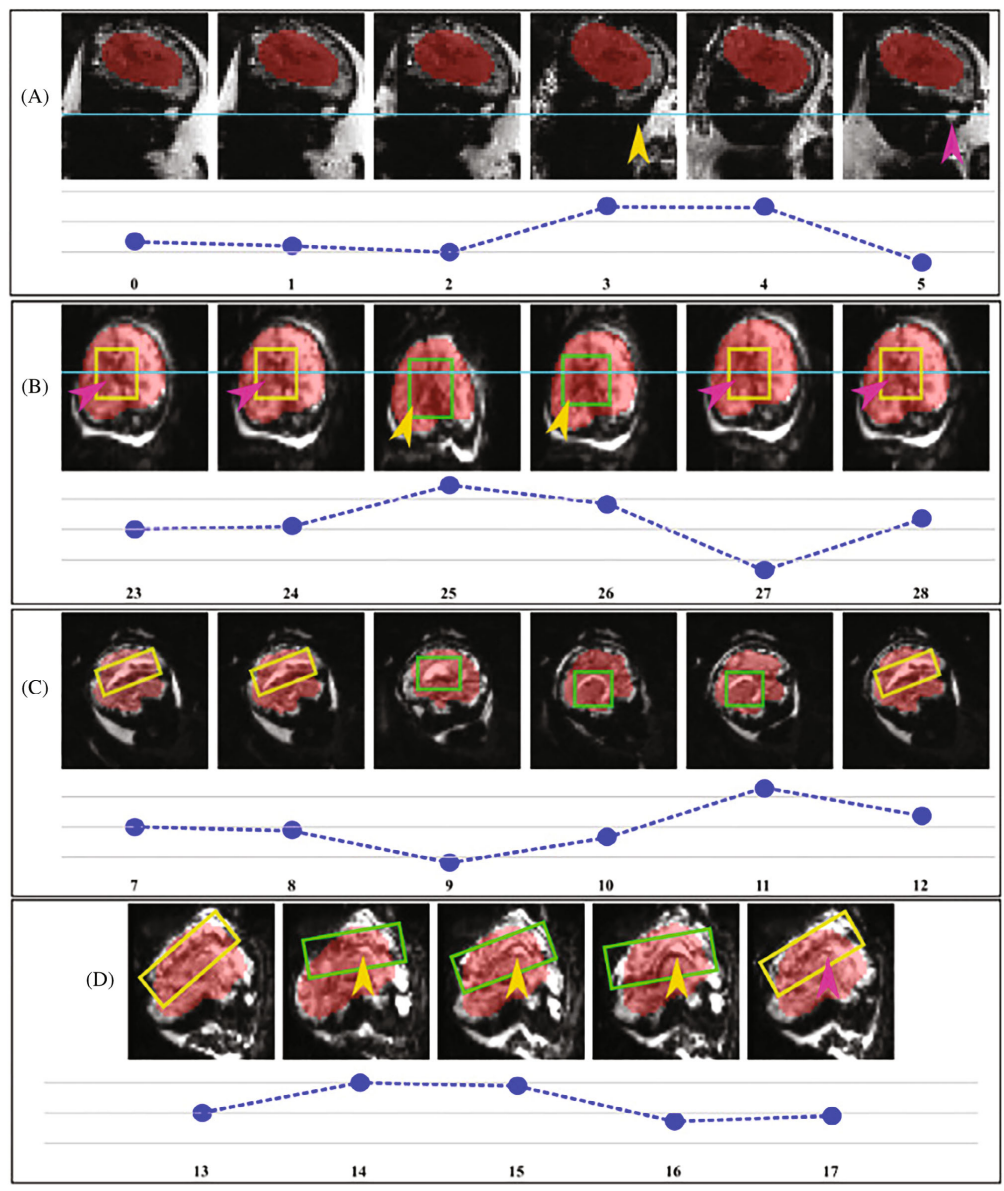
Consecutive repetitions from one plane acquired with real‐time fetal brain motion correction are illustrated for four fetal subjects.

The CoM coordinates extracted from the predicted segmentations during the phantom experiment in Figure 8 show a translational displacement in the x‐coordinate of the segmentation between repetition 6 and 7 (marked with a yellow arrow), followed by an image FOV shift in repetition 9 (purple arrow) that recenters the scan to the moved position of the phantom. FOV adjustments were also observed in repetitions 20, 31, 38, and 48 following the applied motion two repetitions prior. FOV adaptions in the y and z coordinates are further demonstrated. In each case, there is a phantom shift in the FOV and two repetitions later the FOV shifts to match the displacement applied. Figure S2 depicts the corresponding image views for the phantom, with the pink cross marking the calculated CoM of the phantom in repetitions where controlled motion was mimicked and the blue lines marking the FOV center. The shift in the pink cross is detected by the system and two repetitions later a corresponding FOV shift is introduced, with the centre of the phantom overlapping with the centre of the image FOV. Figure 9 shows the translational motion for all 10 fetal subjects.

#### 3.2.2 Fetal experiments

Consecutive repetitions from one plane are illustrated for four fetal subjects in Figure 9 with the corresponding translational motion in x, y, or z directions, depending on the direction where motion predominantly occurred. In A), head translational motion in repetition 3 (yellow arrow) is followed by the corresponding FOV adjustment two repetitions later (pink arrow) that re‐aligns the fetal eye (blue line) to the first three repetitions of the scan. In B), head translation led to a displacement of the centre of the ventricles in repetition 25 (blue line). Additionally, although the same slice location is displayed for all repetitions, the X shape of the ventricles can only be observed in repetitions 25 and 26 (green box, yellow arrow), whereas in repetitions 23 and 24 (yellow box, pink arrow) these are only partially visible. FOV re‐adjustment was applied in repetition 27 (yellow box, pink arrow), with re‐alignment of the center of the ventricles and view identical to repetitions 23 and 24. (C) shows images of a fetus with ventriculomegaly (enlarged ventricles). Motion occurred between repetitions 9 and 11, causing changes in the view of the fluid‐filled midline cavum (green box) and the amount of visible cerebrospinal fluid when compared with repetitions 7 and 8 (yellow box). When the FOV was re‐adjusted, the view of the cavum and cerebrospinal fluid are identical to the first two images. In (D), with fetal head displacements between repetitions 14 and 16 cerebrospinal fluid becomes visible (green box, yellow arrow). With re‐adjustment in repetition 17, fluid is no longer visible (yellow box, pink arrow).

## 4 DISCUSSION

The here presented prospective motion correction approach, forfeiting the need for additional navigator‐type scans by using the EPI read‐outs as intrinsic navigators, allows to follow the fetal motion without any additional time penalty.

While the achieved localization of the fetal brain, evaluated using DSC and IoU, is inferior to published studies focusing on anatomical high‐resolution images,^11^, ^12^, ^13^, ^14^, ^15^, ^17^ this represents one of the first studies enabling this step with intrinsically lower resolution functional data.

The experiment involving the retrospective evaluation of the localization network performance on different data categories—imaging parameters (TE), fetal position, gestational age, and field strength—allowed to draw the following conclusions: (1) although TE2 offered the best performance, the model demonstrated enough robustness to successfully extract the fetal brain even in images of very low contrast and signal; (2) slightly higher performance was observed in the cephalic position, which may be justified by the proximity to the coils and subsequently increased signal and the head restraining in the narrower section of the uterus, however, the model was able to successfully localise the fetal brain independent of its location within the uterus; (3) although slightly lower performance was observed <23 weeks, robustness was again demonstrated by the model across the different GAs and associated different brain volumes and cortical folding patterns; (4) the model, trained on 1.5/3T data, showed great generalization ability when tested on low‐field data.

The assessment of the effect of the computed DSC on the estimation of fetal head motion allowed to conclude the high DSC scores obtained in TE2 allow successful estimation of the fetal head motion, further justifying the use of TE2 for real‐time fetal brain tracking.

The results obtained from the real‐time fetal brain tracking experiments demonstrated the ability of the method to detect changes in the fetal head position and adjust the acquisition geometry accordingly, with an immediate clear enhancement of the data quality observed.

The chosen approach, built on the open‐source Gadgetron framework allows for easy translation and dissemination, all steps including the pretrained network, localization and tracking Gadgets as well as all anonymized fetal data are available to any interested researchers (https://github.com/saranevessilva/fetalbraintracking).

There are, however, some limitations in the currently presented study. First, the number of prospective subjects is limited and this can hence be mainly seen as a proof‐of‐concept study. However, the 10 included subjects, as well as the large training dataset, cover a wide range of gestational ages, including very young fetuses with significant motion (17 weeks gestational age), fetuses in breech presentation as well as examples during uterine contractions. Next, the tracking currently focuses on translations in all three directions but does not yet allow correcting rotations. This constitutes an important next step which will be addressed by extracting the main direction of the mask by, for instance, fitting an ellipsoid model or performing singular value decomposition with rotational parameters of the region of interest extracted. Currently, the correction is applied two repetitions after the fetal motion occurred, thus changes to the architecture might lead to additional speed gains in the future. Furthermore, the current protocol implemented in the scanner for real‐time fetal brain tracking is a BOLD sequence with one echo time, and further efforts will focus on including multi‐echo sequences to allow for quantitative T2* analysis as well as EPI‐based diffusion sequences. As with all prospective methods, quantification of the improvement is difficult. Further efforts in addition to the included phantom studies could include mechanical ventilators or similarly predictable motion phantoms. Lastly, although our aim is to achieve a TR <2 s, the current timing is 11.5 s. TR is limited by the longer T1, by the current latency observed and the use of nonaccelerated EPI. Future low‐field real‐time tracking, as already prepared here by demonstrating the ability of the network to localize the fetal head on low‐field data, could alleviate this concern. Additionally, the current protocol does not use acceleration as the reconstruction task cannot yet be employed in accelerated acquisitions—this is currently being addressed. Future steps additionally include exploring simultaneous multislice acquisitions, which allow a significant reduction of TR.

This study shows that intrinsic, prospective, real‐time motion correction is feasible for functional fetal MRI and presents an open‐source framework open for further extensions. Clear next applications are the quantification of the maternal and fetal BOLD response during hyperoxygenation^33^ and the assessment of oxygenation changes during subclinical uterine contractions in both brain and placenta.^34^ Furthermore, as mentioned above, long connectome‐style diffusion sequences could benefit from the presented improvements.

## Supporting information




**Figure S1.** (A) The various acquisition parameters regarding field strength, resolution and echo time are stated for the two datasets: the 157 fetal subjects scanned at 1.5T/3T and the 29 fetal subjects scanned at 0.55T. These were deliberately diversified to increase the robustness of the fetal brain localization network. (B) Demographics on gestational age at scan, maternal BMI and fetal position are shown and additionally demonstrate the diversity of the training/validation/testing datasets.
**Figure S2.** A sequence of images illustrating motion tracking for the phantom experiment with all three planes depicted. The blue lines mark the center of the image FOV and the pink crosses mark the center‐of‐mass of the phantom in the repetitions where translational displacements were mimicked. The shift in the center‐of‐mass of the phantom is detected by the system and two repetitions later a corresponding FOV shift is introduced, with the phantom returning to the center of the image FOV.
**Figure S3.** Center‐of‐mass coordinates calculated from images of one fetal subject are plotted for the first ten repetitions of the scan in x, y and z directions. The pink line depicts the head motion detection calculated from gold standard brain masks, and the purple, blue and green lines depict the center‐of‐mass of the predicted segmentations from TE1, TE2 and TE3 images, respectively. The mean squared errors calculated for TE1, TE2 and TE3 images were, respectively, [5.64, 545.0, 0.13] ± [9.22, 458.29, 0.097] mm, [0.85, 0.72, 0.47] ± [0.76, 1.24, 0.46] mm and [3.2, 61.14, 0.69] ± [8.08, 182.29, 0.42] mm in x, y and z.
