# Supplementary material for: Real‐time fetal brain tracking for functional fetal MRI
Source: Magn Reson Med. 2023 Jul 19;90(6):2306–20. doi: 10.1002/mrm.29803 (PMC10952752; doi:10.1002/mrm.29803)
Supplement: Supplementary file 1 — Figure S1. (A) The various acquisition parameters regarding field strength, resolution and echo time are stated for the two datasets: the 157 fetal subjects scanned at 1.5T/3T and the 29 fetal subjects scanned at 0.55T. These were deliberately diversified to increase the robustness of the fetal brain localization network. (B) Demographics on gestational age at scan, maternal BMI and fetal position are shown and additionally demonstrate the diversity of the training/validation/testing datasets. Figure S2. A sequence of images illustrating motion tracking for the phantom experiment with all three planes depicted. The blue lines mark the center of the image FOV and the pink crosses mark the center‐of‐mass of the phantom in the repetitions where translational displacements were mimicked. The shift in the center‐of‐mass of the phantom is detected by the system and two repetitions later a corresponding FOV shift is introduced, with the phantom returning to the center of the image FOV. Figure S3. Center‐of‐mass coordinates calculated from images of one fetal subject are plotted for the first ten repetitions of the scan in x, y and z directions. The pink line depicts the head motion detection calculated from gold standard brain masks, and the purple, blue and green lines depict the center‐of‐mass of the predicted segmentations from TE1, TE2 and TE3 images, respectively. The mean squared errors calculated for TE1, TE2 and TE3 images were, respectively, [5.64, 545.0, 0.13] ± [9.22, 458.29, 0.097] mm, [0.85, 0.72, 0.47] ± [0.76, 1.24, 0.46] mm and [3.2, 61.14, 0.69] ± [8.08, 182.29, 0.42] mm in x, y and z. [file MRM-90-2306-s001.pdf]

ORIGINAL ARTICLE

Journal Section

Real-time fetal brain tracking for functional fetal MRI

Sara Neves Silva[1,2] | Jordina Aviles Verdera[1,2] | Raphael Tomi-Tricot[1,2,3] | Radhouene Neji[2,3] | Alena Uus[1,2] | Irina Grigorescu[1,2] | Thomas Wilkinson[1,2] | Valery Ozenne[4] | Alexander Lewin[5,6] | Lisa Story[1,7] | Enrico De Vita[2,8] | Mary Rutherford[1,2] | Kuberan Pushparajah[2] | Jo Hajnal[1,2] | Jana Hutter[1,2]

[1]Centre for the Developing Brain, School of Biomedical Engineering & Imaging Sciences, King's College London, London, UK

[2]Biomedical Engineering Department, School of Biomedical Engineering & Imaging Sciences, King's College London, London, UK

[3]MR Research Collaborations, Siemens Healthcare Limited, Camberley, United Kingdom

[4]CNRS, CRMSB, UMR 5536, IHU Liryc, Université de Bordeaux, Bordeaux, France

[5]Institute of Neuroscience and Medicine 11, INM-11, Forschungszentrum Jülich, Jülich, Germany

[6]RWTH Aachen University, Aachen, Germany

[7]Department of Women & Children's Health, King's College London, London, UK

[8]MRI Physics Group, Great Ormond Street Hospital, London, UK

Correspondence
Sara Neves Silva, King's College London, London, UK
Email: sara.neves_silva@kcl.ac.uk

Purpose: To improve motion robustness of functional fetal MRI scans by developing an intrinsic real-time motion correction method. MRI provides an ideal tool to characterise fetal brain development and growth. It is, however, a relatively slow imaging technique and therefore extremely susceptible to subject motion, particularly in functional MRI experiments acquiring multiple Echo-Planar-Imaging-based repetitions, e.g., diffusion MRI or BOLD MRI.
Methods: A 3D UNet was trained on 125 fetal datasets to track the fetal brain position in each repetition of the scan in real time. This tracking, inserted into a Gadgetron pipeline on a clinical scanner, allows updating the position of the field of view in a modified EPI sequence. The method was evaluated in real-time in controlled-motion phantom experiments and ten fetal MR studies (17+4-34+3 gestational weeks) at 3T. The localization network was additionally tested retrospectively on 29 low-field (0.55T) datasets.
Results: Our method achieved real-time fetal head tracking and prospective correction of the acquisition geometry. Lo-

calization performance achieved Dice scores of 84.4% and 82.3% respectively for both the unseen 1.5T/3T and 0.55T fetal data, with values higher for cephalic fetuses and increasing with gestational age.

Conclusions: Our technique was able to follow the fetal brain even for fetuses under 18 weeks GA in real-time at 3T and was successfully applied "offline" to new cohorts on 0.55T. Next, it will be deployed to other modalities such as fetal diffusion MRI and to cohorts of pregnant participants diagnosed with pregnancy complications, e.g., pre-eclampsia and congenital heart disease.

KEYWORDS

Fetal MRI, motion detection, motion correction, tracking, fetal brain development, T2* relaxometry, BOLD, Diffusion MRI

1 | SUPPLEMENTARY MATERIAL

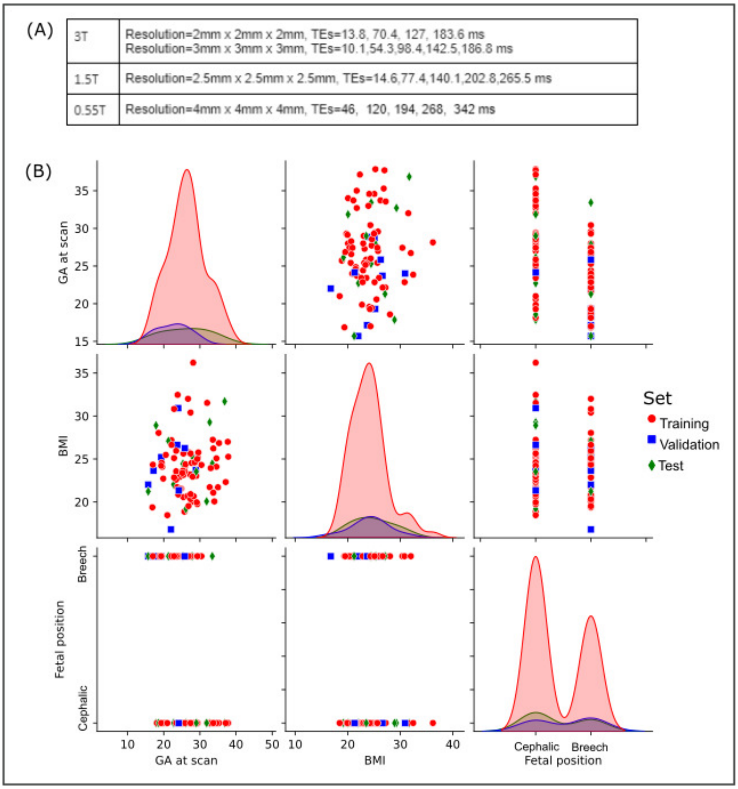

Figure S1: A) The various acquisition parameters regarding field strength, resolution and echo time are stated for the two datasets: the 157 fetal subjects scanned at 1.5T/3T and the 29 fetal subjects scanned at 0.55T. These were deliberately diversified to increase the robustness of the fetal brain localization network. B) Demographics on gestational age at scan, maternal BMI and fetal position are shown and additionally demonstrate the diversity of the training/validation/testing datasets.

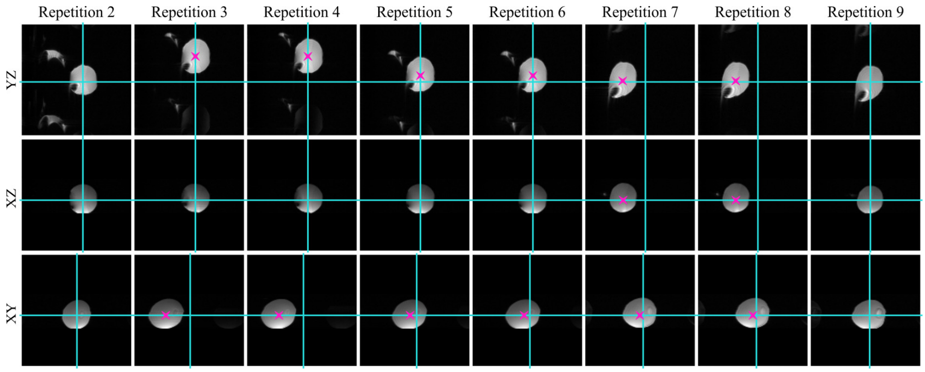

Figure S2: A sequence of images illustrating motion tracking for the phantom experiment with all three planes depicted. The blue lines mark the centre of the image FOV and the pink crosses mark the centre-of-mass of the phantom in the repetitions where translational displacements were mimicked. The shift in the centre-of-mass of the phantom is detected by the system and 2 repetitions later a corresponding FOV shift is introduced, with the phantom returning to the centre of the image FOV.

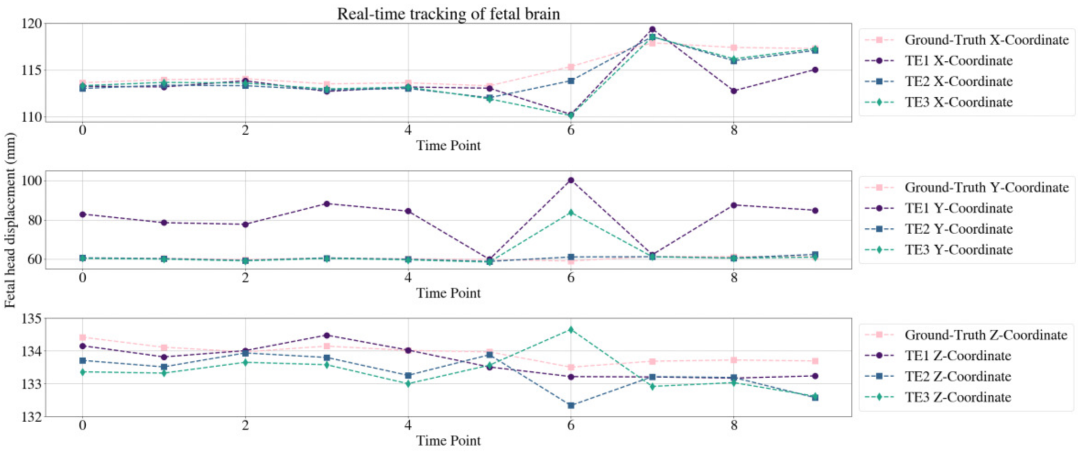

Figure S3: Centre-of-mass coordinates calculated from images of one fetal subject are plotted for the first ten repetitions of the scan in X, Y and Z directions. The pink line depicts the head motion detection calculated from gold standard brain masks, and the purple, blue and green lines depict the centre-of-mass of the predicted segmentations from TE1, TE2 and TE3 images, respectively. The mean squared errors calculated for TE1, TE2 and TE3 images were, respectively, [5.64, 545.0, 0.13]±[9.22, 458.29, 0.097]mm, [0.85, 0.72, 0.47]±[0.76, 1.24, 0.46]mm and [3.2, 61.14, 0.69]±[8.08, 182.29, 0.42]mm in X, Y and Z.
